# Energy-efficient bifunctional CoB_*x*_/GDY catalyst for urea-assisted hydrogen production *via* electrochemical urea oxidation and hydrogen evolution

**DOI:** 10.1039/d5ra06956d

**Published:** 2025-10-29

**Authors:** Teng Liu, Ting Wang, Hao Niu, Chunli Wang, Zhenwei Wei, Jingjing Wang, Xuepeng Yin, Shanmin Gao

**Affiliations:** a School of Chemistry & Chemical Engineering, Linyi University Linyi 276000 PR China yinxuepeng@lyu.edu.cn; b School of Chemical Engineering and Technology, Tianjin University Tianjin 300072 PR China weizw@tju.edu.cn; c College of Biological and Chemical Engineering, Qilu Institute of Technology Jinan 250200 PR China

## Abstract

Efficient and durable bifunctional electrocatalysts for the urea oxidation reaction (UOR) and hydrogen evolution reaction (HER) are vital for sustainable hydrogen production coupled with wastewater remediation. Herein, we present a CoB_*x*_/GDY catalyst electrode that delivers outstanding activity and stability for both reactions. CoB_*x*_/GDY requires only 1.41 V *vs.* RHE to achieve 50 mA cm^−2^ for UOR, while for HER it exhibits an overpotential of 118 mV at 10 mA cm^−2^ with a Tafel slope of 97.2 mV dec^−1^. The superior performance is attributed to the conductive π-conjugated GDY framework, which provides abundant accessible active sites and facilitates rapid electron transfer. These results highlight CoB_*x*_/GDY as a promising bifunctional catalyst for energy-efficient hydrogen production from urea-rich wastewater and offer a general strategy for designing advanced transition-metal/graphdiyne hybrid electrocatalysts.

## Abstract 2

The CoB_*x*_/GDY electrode delivers exceptional UOR and HER performance, enabled by the π-conjugated GDY framework that ensures high conductivity, abundant active sites, and fast electron transfer.

## Graphical abstract

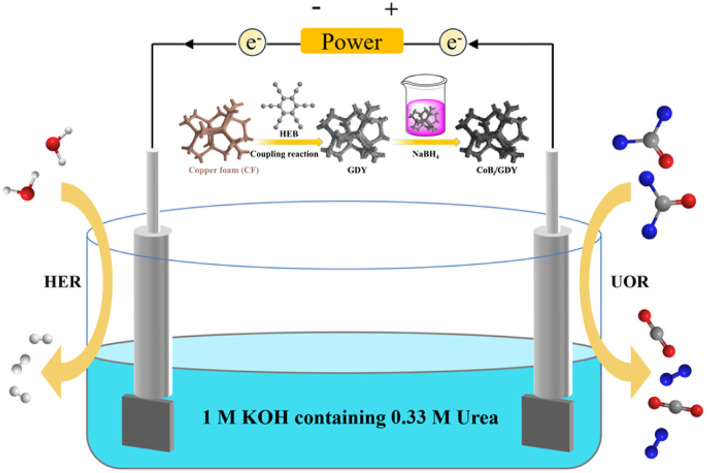

## Introduction

With the continuous increase in global energy demand and heightened environmental awareness, the development of clean and renewable energy systems has become a critical challenge for the international community. Among various alternatives, hydrogen, which is characterized by its high energy density and zero emissions, is considered as a pivotal component of future energy infrastructures.^1^ Nevertheless, the large-scale deployment of hydrogen technologies continues to face significant challenges, with the efficient and cost-effective production of hydrogen remaining a critical bottleneck. Water electrolysis, which utilizes renewable energy sources such as solar and wind power to directly produce hydrogen, offers notable advantages, including environmental sustainability and high operational flexibility.^2^ Although the theoretical cell voltage for water electrolysis is 1.23 V, practical operation typically requires 1.8–2.6 V, underscoring the energy-intensive nature of the process.^3,4^ In conventional water electrolysis, the oxygen evolution reaction (OER) during water oxidation, due to its inherently high overpotential, is a major factor limiting overall energy efficiency.^5,6^ This high overpotential not only increases energy consumption but also compromise catalyst stability, thereby reducing the service life of the electrolyzer.

A potential strategy to address this challenge is to replace, or partially replace, the oxygen evolution reaction (OER) with alternative anodic reactions that occur at lower overpotentials. Representative examples include electrocatalytic alcohol oxidation, hydrazine oxidation, urea oxidation, and glucose oxidation, among others, all of which can facilitate water electrolysis by reducing the overpotential.^7–10^ Among these, the electrochemical urea oxidation reaction (UOR) is particularly attractive because its thermodynamic equilibrium potential (0.37 V *vs.* RHE) is substantially lower than that of the OER, enabling low-energy hydrogen production when integrated into water electrolysis.^9^ In addition to enhancing hydrogen production efficiency and economic viability, this strategy addresses pressing environmental concerns: large-scale industrial wastewater discharge and the extensive use of urea-based fertilizers have resulted in excessive nitrogen loading in water bodies, driving eutrophication and threatening aquatic ecosystems.^11^ By simultaneously treating urea-rich wastewater and generating hydrogen, the UOR offers the dual benefits of pollution mitigation and resource valorization. Nevertheless, the UOR involves a six-electron transfer process and the adsorption–desorption of complex intermediates, leading to sluggish kinetics and poor catalyst stability.^12^ Therefore, the rational design of efficient, durable, and kinetically favorable electrocatalysts is crucial for promoting the large-scale application of electrocatalytic urea oxidation.

The exploration and development of efficient catalysts for urea oxidation have become a central focus in electrocatalysis research. These catalysts can lower the reaction barrier, enhance catalytic efficiency, and accelerate the kinetics of the UOR process.^12^ Noble metals, such as Pt, have demonstrated superior electrocatalytic activity toward the UOR due to their intrinsically low overpotentials.^13^ However, the high cost and scarcity of noble metals significantly limit their large-scale industrial implementation. Consequently, research efforts have increasingly focused on cost-effective and earth-abundant transition-metal-based catalysts. These materials exhibit promising UOR activity, primarily due to their tunable coordination environments and adjustable d-orbital configurations.^14^ Despite these advantages, transition-metal-based catalysts still face several critical challenges. One of the foremost challenges is their catalytic activity. Although they exhibit measurable activity, the reaction rates remain insufficient to meet the demands of practical operating conditions. Accordingly, strategies to enhance their intrinsic activity are essential for improving both catalytic efficiency and economic feasibility. Another major limitation is their durability under operational conditions. In the harsh electrochemical environment of the UOR, characterized by high current densities and repeated potential cycling, catalyst degradation and performance deterioration are frequently observed.^15^ Therefore, achieving long-term stability and reliability is a prerequisite for the sustainable and efficient application of transition-metal-based catalysts in urea oxidation.

To address these challenges and advance practical applications, various design and synthesis strategies have been proposed to enhance both the catalytic activity and durability of transition-metal-based catalysts. Current approaches to developing efficient urea oxidation catalysts can be broadly categorized into three strategies: (i) increasing the density of active sites, (ii) enhancing intrinsic catalytic activity, and (iii) improving catalyst dispersion while minimizing interfacial resistance.^16^ In 2023, Wang Dingsheng and Li Yadong introduced an interfacial chemical modulation strategy by constructing a two-dimensional/two-dimensional Ru–Co dual-atomic-site (DAS)-modified NiO heterostructure (Ru–Co DAS/NiO), which exhibited remarkable activity and durability for electrocatalytic UOR.^17^ In the same year, Myong Yong Choi and co-workers reported a Mo-doping approach to modulate Ni/NiO nanocomposites. The introduction of Mo promoted the oxidation of Ni^2+^ to Ni^3+^, thereby generating abundant active sites.^18^ As a result, urea-assisted water splitting required only 1.45 V to achieve a current density of 10 mA cm^−2^.

In this work, we present a novel CoB_*x*_/graphdiyne (CoB_*x*_/GDY) bifunctional catalyst electrode exhibiting high activity and durability for both the urea oxidation reaction (UOR) and the hydrogen evolution reaction (HER). Graphdiyne (GDY), a two-dimensional carbon allotrope composed of benzene rings connected by diacetylene linkages, was selected as the support material due to its electron-rich, π-conjugated network, uniformly distributed acetylenic bonds, and strong affinity for anchoring metal species.^19^ These features not only facilitate rapid charge transfer but also provide abundant coordination sites for stabilizing catalytic nanoparticles and tuning their electronic structures. Benefiting from these advantages, CoB_*x*_ species were uniformly anchored onto GDY *via* a facile hydrothermal synthesis, yielding the CoB_*x*_/GDY catalyst. CoB_*x*_/GDY exhibited superior UOR performance, requiring only 180 mV to achieve a current density of 50 mA cm^−2^ in 1.0 M KOH containing 0.33 M urea. Moreover, the catalyst displayed excellent HER activity, enabling its use as a bifunctional electrocatalyst for UOR-assisted hydrogen production. The incorporation of GDY significantly enhanced the catalytic performance of CoB_*x*_ by providing abundant active sites and a rich electron environment, thereby promoting both the UOR and HER processes. This work highlights the potential of CoB_*x*_/GDY as an efficient bifunctional electrocatalyst and proposes a strategy for designing advanced catalysts for urea oxidation and related energy-conversion applications.

## Experimental section

### Materials

Cobalt nitrate hexahydrate (Co(NO_3_)_2_·6H_2_O, AR grade), sodium borohydride (NaBH_4_, AR grade), ruthenium(iv) oxide (RuO_2_, 99%, AR grade), and urea (CO(NH_2_)_2_, AR grade) were purchased from Adamas Reagent Co., Ltd. Commercial 20% Pt/C was purchased from Hesen Chemical Reagent. All chemicals were of analytical grade and used without further purification. Copper foam (CF) substrates were cleaned by ultrasonic treatment in 3 M HCl and acetone for 5 minutes, respectively. Ultrapure water with a resistivity of 18.2 MΩ cm was used throughout all experiments. Graphdiyne (GDY) nanowalls were synthesized in-house *via* a cross-coupling reaction of hexaethynylbenzene (HEB), following a previously reported procedure with minor modifications.^20^

### Synthesis

GDY nanowalls on CF (1 cm × 0.5 cm) were used as the substrate for CoB_*x*_ deposition. The GDY/CF electrode was first immersed in an aqueous solution of cobalt nitrate hexahydrate (Co(NO_3_)_2_·6H_2_O, 40 mM) at room temperature for 2 h to allow sufficient adsorption of Co^2+^ ions. Subsequently, sodium borohydride (NaBH_4_, 0.5 M) solution was added dropwise to initiate the reduction reaction, and the mixture was maintained for 40 min under ambient conditions. After the reaction, the electrode was thoroughly rinsed with ultrapure water and stored under vacuum. The resulting sample is denoted as CoB_*x*_/GDY.

For comparison, CoB_*x*_ was also synthesized directly on bare CF substrates using the same procedure, without the presence of GDY nanowalls. The obtained sample is referred to as CoB_*x*_.

### Characterization

Scanning electron microscopy (SEM) images were obtained using a Phenom XL system from Guoyi Quantum Co., Ltd. Focused ion beam scanning electron microscopy (FIB-SEM) was performed on a Helios NanoLab 460HP system. Transmission electron microscopy (TEM) was carried out using a Tecnai G2 Spirit TWIN at an acceleration voltage of 120 kV. X-ray photoelectron spectroscopy (XPS) measurements were conducted on an ESCALAB 250Xi system. Raman spectra were collected using a high-resolution laser confocal fiber Raman spectrometer (HORIBA EVOLUTION, HORIBA Jobin Yvon, France) with a 532 nm excitation wavelength. X-ray diffraction (XRD) patterns were recorded on a Rigaku diffractometer (Rigaku Corporation, Japan). Electrochemical measurements were performed using a CHI 760E electrochemical workstation (Shanghai Chenhua Instrument Co., Ltd).

### Electrochemical measurements

Electrocatalytic measurements for urea oxidation reaction (UOR) and hydrogen evolution reaction (HER) were carried out using a CHI 760E electrochemical workstation (Shanghai Chenhua Instrument Co., Ltd) in a standard three-electrode configuration. CoB_*x*_/GDY and CoB_*x*_ electrodes were employed as the working electrodes, while a carbon rod and an Ag/AgCl (saturated KCl) electrode served as the counter and reference electrodes, respectively. All potentials were converted to the reversible hydrogen electrode (RHE) scale using the equation: *E*(RHE) = *E*(Ag/AgCl) + 0.198 + 0.059 × pH. For UOR measurements, 1.0 M KOH containing 0.33 M urea was used as the electrolyte. For HER measurements, 1.0 M KOH was used. Prior to electrochemical testing, the electrodes were activated by 20 consecutive cyclic voltammetry (CV) scans at a scan rate of 100 mV s^−1^ to ensure stability. Linear sweep voltammetry (LSV) was performed at a scan rate of 5 mV s^−1^ to evaluate the electrocatalytic activity. Commercial Pt/C (20 wt%) and RuO_2_ were used as reference catalysts for HER and UOR, respectively. Catalyst inks were prepared by dispersing 9.0 mg of Pt/C or RuO_2_ into 2.5 mL of ethanol/water mixture (v/v = 1 : 4) containing 100 μL of 5 wt% Nafion solution, followed by sonication for 1 h. Subsequently, 140 μL of Pt/C ink or 28 μL of RuO_2_ ink was drop-cast onto CF substrates (loading amount: 400 μg cm^−2^), respectively.

## Results and discussion

**Scheme 1:**
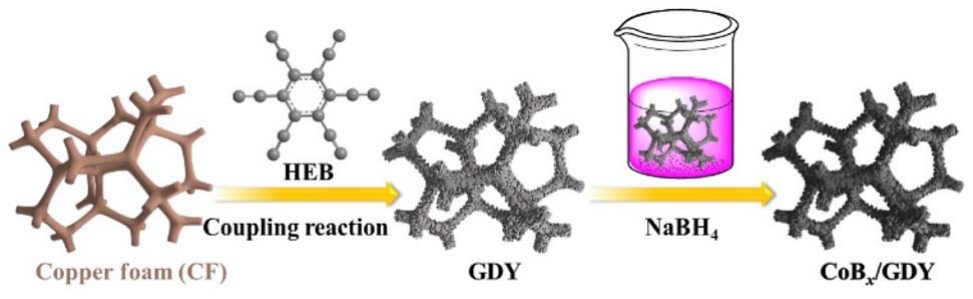
The synthesis of CoB_*x*_ nanosheets grown *in situ* on GDY nanowalls at room temperature.

In this study, CoB_*x*_ nanosheet/graphdiyne (GDY) heterostructures were fabricated, as illustrated in Scheme 1. GDY nanowalls were grown directly on copper foam (CF) *via* the cross-coupling reaction of hexaethynylbenzene (HEB), following the procedure reported by Zhang *et al.*^20^ After thorough washing to remove residual reactants and oligomers, the as-prepared GDY nanosheets served as a conductive, high-surface-area substrate for subsequent CoB_*x*_ deposition. The cobalt precursor (Co^2+^) was then converted *in situ* into CoB_*x*_ on the GDY surface through a wet-chemical reduction with NaBH_4_ in aqueous solution, yielding a CoB_*x*_/GDY heterojunction catalyst. For comparison, a pure CoB_*x*_ sample was synthesized under identical conditions but without GDY.

**Fig. 1:**
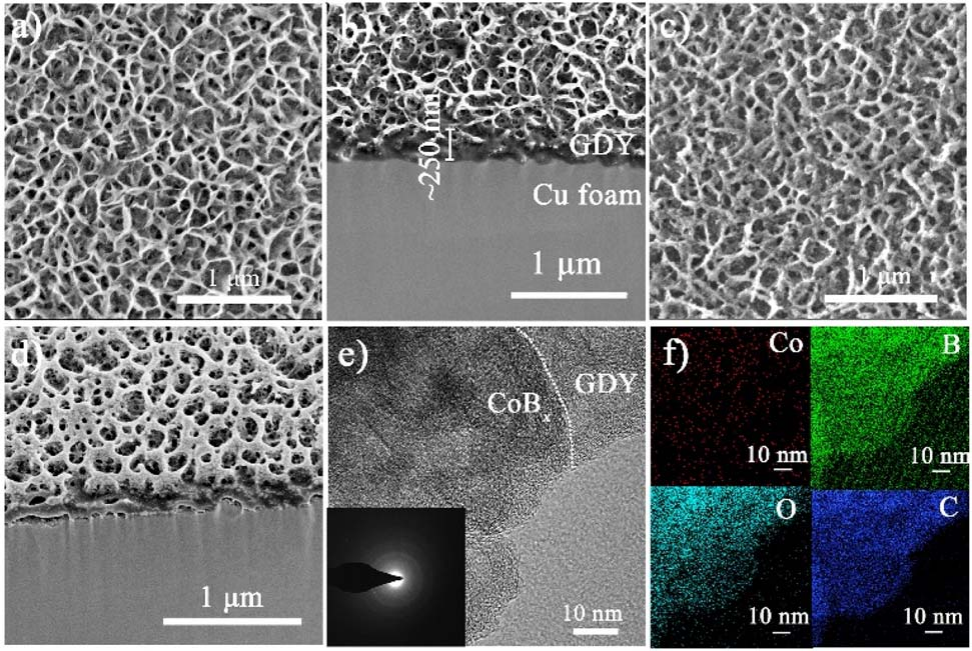
(a) SEM image of GDY. (b) FIB-SEM image of GDY. (c) SEM image of CoB_*x*_/GDY. (d) FIB-SEM image of CoB_*x*_/GDY. (e) TEM image of CoB_*x*_/GDY, with the inset showing the corresponding selected area electron diffraction (SAED) image of CoB_*x*_/GDY. (f) EDS mapping of CoB_*x*_/GDY showing the distribution of Co (red), B (green), O (cyan), and C (blue).

The morphology, structure, and spatial distribution of the as-prepared GDY nanowalls and CoB_*x*_/GDY heterostructures were characterized by scanning electron microscopy (SEM), transmission electron microscopy (TEM), and elemental mapping. As shown in Fig. 1a, the GDY nanowalls are vertically aligned on the CF substrate, forming a continuous, interconnected porous network. The corresponding FIB-SEM image (Fig. 1b) reveals that the GDY layer has an average thickness of approximately 250 nm and is tightly anchored to the underlying CF, indicating strong interfacial adhesion between GDY and the metallic substrate. For the CoB_*x*_/GDY heterostructures, the SEM image (Fig. 1c) shows that additional nanosheets are uniformly deposited onto the GDY nanowalls. The FIB-SEM image (Fig. 1d) further confirms that these CoB_*x*_ nanosheets intimately coat the GDY framework, conformally covering its surface while maintaining the vertical alignment of the underlying GDY skeleton. The cross-sectional view also indicates that the CoB_*x*_ layer is continuous and well-integrated with the GDY, without observable delamination or voids at the interface. TEM analysis (Fig. 1e and S1) confirms that the CoB_*x*_ nanosheets were *in situ* grown on the GDY substrate, forming a coherent CoB_*x*_/GDY heterojunction. The TEM results also reveal that both the CoB_*x*_ and GDY components are amorphous, as evidenced by the diffuse diffraction rings in the selected-area electron diffraction (SAED) pattern (inset of Fig. 1e). Elemental mapping (Fig. 1f) demonstrates the homogeneous distribution of Co (red), B (green), O (cyan), and C (blue) throughout the CoB_*x*_/GDY structure. These results collectively confirm the successful fabrication of the CoB_*x*_/GDY heterojunction with strong interfacial contact, which is expected to facilitate efficient charge transfer during catalytic processes.

**Fig. 2:**
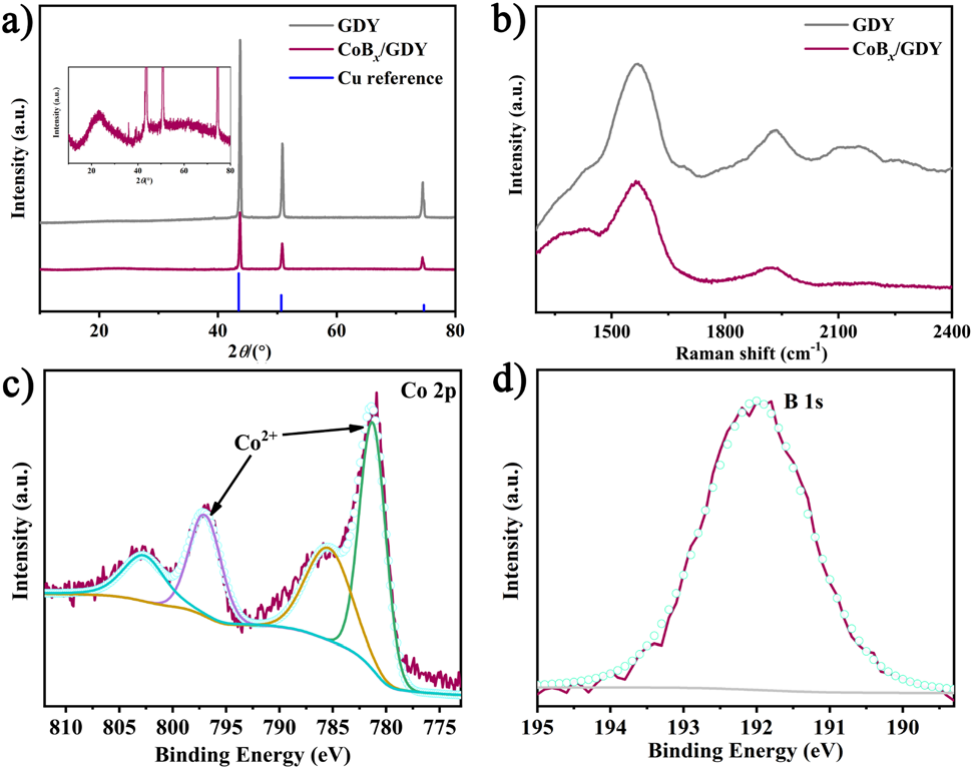
(a) XRD patterns of GDY and CoB_*x*_/GDY, (b) Raman spectra of GDY and CoB_*x*_/GDY, (c) Co 2p, and (d) B 1s XPS patterns of CoB_*x*_/GDY.

The structure and chemical composition of CoB_*x*_/GDY were further examined using powder X-ray diffraction (PXRD), Raman spectroscopy, and X-ray photoelectron spectroscopy (XPS). As shown in Fig. 2a, the PXRD patterns of the as-prepared CoB_*x*_/GDY and pure GDY display diffraction peaks originating solely from the polycrystalline Cu substrate (JCPDS card no. 65-9743, Fig. S2). Even when the PXRD profile of CoB_*x*_/GDY is magnified for closer inspection, no discernible peaks corresponding to GDY or CoB_*x*_ phases are observed, indicating that both CoB_*x*_/GDY and GDY are amorphous, consistent with the TEM and SAED results reported earlier.^21–23^ The Raman spectra (Fig. 2b) exhibit four distinct peaks at approximately 1381, 1568, 1929, and 2155 cm^−1^. Among these, the bands at ∼1381 and ∼1568 cm^−1^ correspond to the characteristic D and G modes of carbon materials, arising from the defect-induced breathing vibration of sp^2^ carbon rings (D) and the in-plane *E*_2g_ vibration of sp^2^ C–C bonds (G), respectively. The peaks at ∼1929 and 2155 cm^−1^ are assigned to the stretching vibrations of alkyne (C≡C) bonds, consistent with the presence of conjugated diyne linkages (–C≡C–C≡C–). These assignments are in good agreement with literature values for similar carbon-based systems.^24^ The decreased intensity of the –C≡C–C≡C– Raman bands following CoB_*x*_ incorporation can be ascribed to the coordination interaction between Co^2+^ ions and the acetylene moieties. This interaction modifies the electronic environment of the diyne units, thereby diminishing their characteristic vibrational response.^23,25^ To further elucidate the chemical states and bonding environments, XPS analysis was conducted. The survey spectrum (Fig. S3) confirms the presence of Co, B, O, and C, consistent with the EDS mapping results. The high-resolution Co 2p spectrum (Fig. 2c) displays two peaks at 781.2 eV and 797.0 eV, corresponding to Co 2p_3/2_ and Co 2p_1/2_, respectively, indicative of Co^2+^ species.^21^ In the B 1 s regions (Fig. 2d), a sharp peak at 192.0 eV is assigned to B^3+^ in borate environments.^21^ Based on quantitative XPS analysis, the atomic ratio of Co : B : O in CoB_*x*_/GDY is approximately 1.50 : 1 : 4.82. Considering the contribution of surface-adsorbed oxygen, the empirical formula of the CoB_*x*_ component can be approximated as Co_3_B_2_O_6_. Moreover, upon hybridization with GDY, a slight decrease in the binding energy is observed (Fig. S5), suggesting that the electron-rich GDY transfers electrons to CoB_*x*_, thereby inducing a subtle reduction in the oxidation state of cobalt. Taken together, these results confirm the successful synthesis of a CoB_*x*_/GDY hybrid material composed of amorphous cobalt-based borate nanosheets intimately integrated with GDY. The observed structural features and interfacial contact suggest that this material merits further investigation for potential application in the urea oxidation reaction (UOR).

**Fig. 3:**
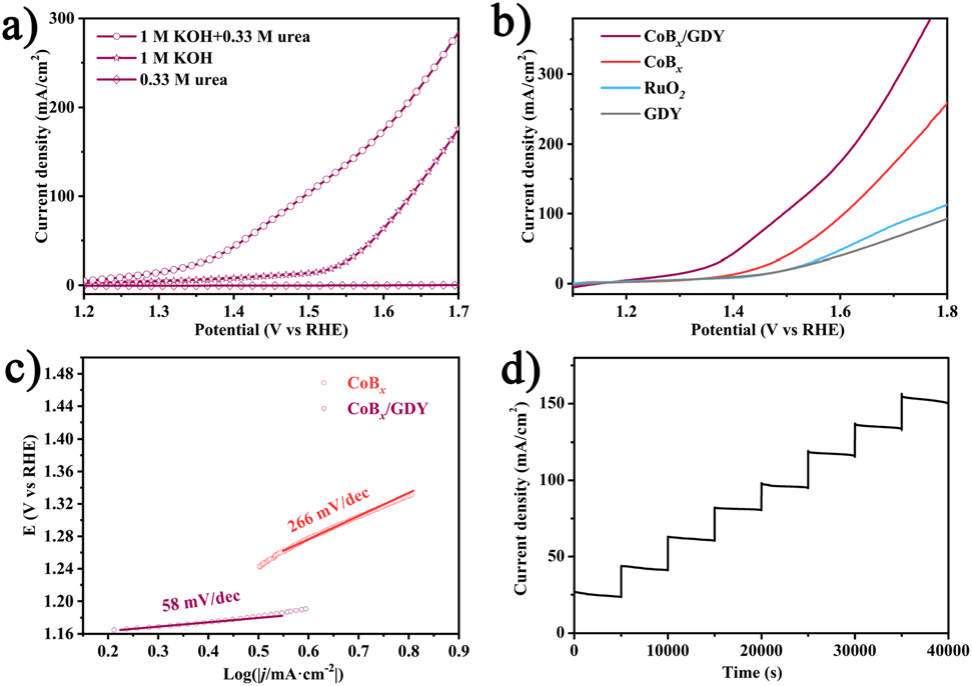
Urea oxidation catalytic performance: (a) LSV curves of CoB_*x*_/GDY in 1.0 M KOH with 0.33 M urea, 1.0 M KOH, and 0.33 M urea with a scan rate of 5 mV s^−1^ (b) LSV curves of CoB_*x*_/GDY, CoB_*x*_, and RuO_2_ in 1.0 M KOH with 0.33 M urea with a scan rate of 5 mV s^−1^. (c) Tafel plots for CoB_*x*_/GDY and CoB_*x*_. (d) Multi-current process of CoB_*x*_/GDY.

The electrocatalytic performance of CoB_*x*_/GDY for the UOR was evaluated under various electrolytic conditions using a typical three-electrode setup (scan rate: 5 mV s^−1^; Fig. 3a). In 1.0 M KOH without urea, CoB_*x*_/GDY exhibits an anodic current density of 50 mA cm^−2^ at 1.59 V *vs.* RHE, corresponding to oxygen evolution reaction (OER) activity. In contrast, negligible current is observed in 0.33 M urea without KOH, suggesting that the UOR does not proceed under alkaline-deficient conditions. Upon the addition of 0.33 M urea to 1.0 M KOH, the anodic current increases significantly, with CoB_*x*_/GDY requiring only 1.41 V *vs.* RHE to reach 50 mA cm^−2^ and 1.49 V *vs.* RHE to reach 100 mA cm^−2^. These results indicate that CoB_*x*_/GDY exhibits promising electrocatalytic activity toward the UOR, with reduced overpotential and enhanced current density. The enhanced performance in alkaline urea solution may be attributed to the increased pH and the availability of hydroxide ions (OH^−^), which act as essential reactants and charge carriers in the UOR pathway. This is consistent with previous studies that highlight the role of OH^−^ in facilitating urea decomposition and electron transfer under alkaline conditions. Fig. 3b compares the UOR performance of CoB_*x*_/GDY, CoB_*x*_, and RuO_2_ supported on CF in 1.0 M KOH containing 0.33 M urea. CoB_*x*_ alone achieves 100 mA cm^−2^ at 1.61 V, outperforming RuO_2_ (*E*_100 mA cm^−2^_ = 1.76 V) under identical conditions. Upon integration with GDY, the CoB_*x*_/GDY hybrid reaches the same current density at a lower potential of 1.49 V, indicating enhanced catalytic efficiency. This enhancement may arise from synergistic effects between CoB_*x*_ and GDY, including improved charge transport, an increased active surface area, and stronger interfacial interactions.

To further evaluate the electrocatalytic kinetics of CoB_*x*_/GDY and CoB_*x*_ electrodes toward the UOR, Tafel analysis was performed (Fig. 3c). The CoB_*x*_/GDY electrode exhibits a Tafel slope of 58 mV dec^−1^, markedly lower than that of CoB_*x*_ (266 mV dec^−1^), indicating more favorable reaction kinetics and faster charge transfer during the UOR. The long-term stability of CoB_*x*_/GDY was evaluated *via* multi-step chronopotentiometry in 1.0 M KOH containing 0.33 M urea (Fig. 3d). The current density was gradually increased from 30 to 150 mA cm^−2^ in increments of ∼20 mA cm^−2^ every 5000 s. Throughout the test, the electrode maintained stable potential responses at each current step, demonstrating excellent durability under prolonged operation and variable current loads. Electrochemical impedance spectroscopy (EIS) was performed to further investigate the charge-transfer characteristics of the catalysts.^26,27^ As shown in the Nyquist plots (Fig. S6), a high-frequency semicircle corresponding to the charge-transfer resistance (*R*_ct_) is observed under the applied potential, indicating the involvement of charge-transfer processes. Under these conditions, CoB_*x*_/GDY displays the smallest semicircle diameter and a lower intercept on the real axis compared with pristine CoB_*x*_ and GDY, corresponding to reduced solution resistance (*R*_s_) and a significantly decreased *R*_ct_. These results reveal that CoB_*x*_/GDY possesses superior electrical conductivity and faster interfacial electron-transfer kinetics, accounting for its enhanced electrocatalytic performance. Taken together, these findings indicate that CoB_*x*_/GDY holds promise for urea-assisted electrolysis and may contribute to energy-efficient hydrogen generation from urea-rich wastewater.

**Fig. 4:**
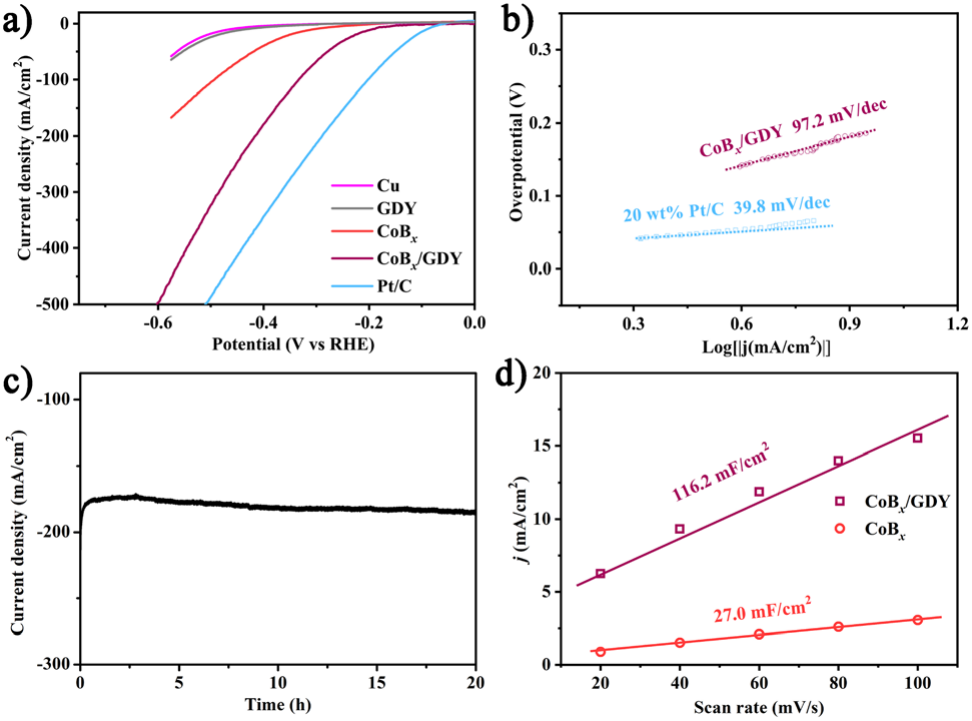
HER catalytic performance: (a) LSV curves of CoB_*x*_/GDY, CoB_*x*_, Pt/C, CF, and GDY in 1.0 M KOH with a scan rate of 5 mV s^−1^. (b) Tafel plots for CoB_*x*_/GDY and CoB_*x*_. (c) Time-dependent current density curve of CoB_*x*_/GDY in 1.0 M KOH. (d) Equivalent double-layer capacitance for CoB_*x*_/GDY and CoB_*x*_.

Recent advancements in alkaline-effective media have significantly enhanced electrocatalytic hydrogen production, mitigating the corrosion issues associated with acidic conditions in electrolyzers. We investigated the hydrogen evolution reaction (HER) performance of the synthesized products, which is crucial for the water-electrolysis-based hydrogen production industry due to the high energy consumption caused by slow kinetics and high overpotential in alkaline environments. For comparative analysis, commercial Pt/C, CoB_*x*_, GDY, and the carrier material CF were selected as reference catalysts. Fig. 4a presents the polarization curves of these catalysts at a scan rate of 5 mV s^−1^ in 1.0 M KOH without iR-compensation, obtained *via* linear sweep voltammetry. As expected, the commercial Pt/C (20 wt% platinum on carbon black) catalyst exhibited the best electrocatalytic performance, requiring only 35 mV of overpotential to achieve a current density of 10 mA cm^−2^. The CoB_*x*_/GDY catalyst exhibited remarkable HER activity, with a minimal overpotential of approximately 118 mV. In contrast, the control samples CoB_*x*_, GDY, and CF exhibited inferior HER activity, with overpotentials exceeding 300 mV. This indicates that the active sites within the CoB_*x*_/GDY composite are essential for the HER process. Notably, GDY and CF showed negligible activity toward HER, likely because the extensive specific surface area of GDY enhances the density of active sites in CoB_*x*_/GDY and improves its electron transport efficiency. This observation is consistent with findings reported in the literature.^5,23^Fig. 4b shows the Tafel plots, with the linear portions fitted using the Tafel equation (*η* = *b* log *j* + *a*, where *j* is the current density and *b* is the Tafel slope), yielding Tafel slopes of 39.8 and 97.2 mV dec^−1^ for commercial Pt/C and CoB_*x*_/GDY catalysts, respectively. The Tafel slope of CoB_*x*_/GDY is slightly larger than commercial Pt/C. A much smaller Tafel slope suggests a higher reaction rate and more favorable kinetics for the catalytic reaction. Additionally, we assessed the long-term electrolysis durability at a fixed overpotential. Fig. 4c demonstrates that the current density stabilized during a prolonged 20-hours test at a constant overpotential of 400 mV, indicating the exceptional durability of the CoB_*x*_/GDY composite for HER.

Cyclic voltammetry (CV) was employed to further evaluate the electrochemical performance of the catalysts. As shown in Fig. 4d and S7, CV measurements were carried out within the non-faradaic potential region to probe the electrochemical double-layer capacitance (*C*_dl_). The CoB_*x*_/GDY exhibits a *C*_dl_ of 116.2 mF cm^−2^, approximately four times higher than that of pristine CoB_*x*_ (27.0 mF cm^−2^), indicating a larger electrochemically active surface area. To assess the structural stability, XPS analysis of the Co 2p and B 1s regions was conducted before and after long-term electrochemical testing (Fig. S8a and b). The spectra display no noticeable shifts in peak positions or intensity ratios, indicating that the chemical state of Co and B remains largely unaltered during operation. Furthermore, FIB-SEM characterization of the post-tested sample shows that the overall morphology is well retained, and Co, B, and other constituent elements remain uniformly distributed, confirming the absence of phase segregation or elemental loss (Fig. S8c and d). This structural robustness under operating conditions underscores the excellent durability of CoB_*x*_/GDY. This stability in surface composition further supports the durability of CoB_*x*_/GDY under electrocatalytic conditions. Overall, the synergistic integration of CoB_*x*_ with graphdiyne endows the catalyst with high intrinsic activity, rapid charge-transfer kinetics, and robust structural stability, collectively underpinning its outstanding bifunctional electrocatalytic performance. Given the favorable performance of CoB_*x*_/GDY toward both the UOR and HER, we further evaluated its overall electrocatalytic activity by employing CoB_*x*_/GDY as both the anode and cathode (Fig. S9). At a current density of 10 mA cm^−2^, the CoB_*x*_/GDY∥CoB_*x*_/GDY cell requires a voltage of only 1.42 V, which is lower than that of the benchmark Pt/C∥RuO_2_ couple (1.45 V). Moreover, with increasing applied voltage, the current density of the CoB_*x*_/GDY∥CoB_*x*_/GDY cell rises more steeply, underscoring its superior bifunctional catalytic capability. To place our findings in context, we conducted a comparative analysis of previously reported UOR catalysts based on Ni and other transition metals. Numerous studies have demonstrated that Ni-based catalysts generally deliver the most outstanding UOR performance, owing to their favorable electronic structure and strong affinity for urea oxidation intermediates (Table S1). In contrast, catalysts constructed from other metals such as Co, Cu, Fe, and Mo typically exhibit lower activity. Within this framework, the CoB_*x*_/GDY hybrid developed in the present work displays relatively high UOR activity, surpassing many of the reported non-Ni systems and even approaching or exceeding that of certain Ni-based catalysts., thereby highlighting its promise as an efficient bifunctional electrocatalyst. Overall, these findings not only validate the effectiveness of the CoB_*x*_/GDY architecture for urea-assisted hydrogen production but also provide valuable design insights for next-generation transition-metal/carbon hybrid catalysts.

## Conclusions

In summary, a CoB_*x*_/GDY hybrid electrocatalyst was successfully fabricated and evaluated for both the urea oxidation reaction (UOR) and the hydrogen evolution reaction (HER). The catalyst delivers outstanding UOR performance, requiring only 1.41 V *vs.* RHE to achieve 50 mA cm^−2^, and exhibits excellent HER activity with an overpotential of 118 mV at 10 mA cm^−2^, a Tafel slope of 97.2 mV dec^−1^, and superior long-term stability. These bifunctional catalytic properties are attributed to the synergistic interaction between CoB_*x*_ and GDY, which enhances charge-transfer efficiency and increases the density of accessible active sites. This work demonstrates the significant potential of CoB_*x*_/GDY for energy-efficient hydrogen production *via* urea-assisted electrolysis and offers a general strategy for designing advanced transition-metal/graphdiyne hybrid catalysts for sustainable energy conversion and environmental remediation.

## Author contributions

Teng Liu, Ting Wang, and Jingjing Wang: writing – original draft, investigation, data curation, validation. Hao Niu and Chunli Wang: investigation, and validation, writing – review & editing. Xuepeng Yin, Zhenwei Wei, and Shanmin Gao: investigation, conceptualization, supervision, project administration, writing – review & editing. All authors discussed the results and provided input to the manuscript.

## Conflicts of interest

There are no conflicts to declare.

## Supplementary Material

RA-015-D5RA06956D-s001

## Data Availability

The raw data presented in this work and the fitting results are available at Figshare *via* the following link: https://figshare.com/account/articles/30121210. Supplementary information: the data supporting the findings of this study, “Energy-efficient bifunctional CoB*_x_*/GDY catalyst for urea-assisted hydrogen production *via* electrochemical urea oxidation and hydrogen evolution”, are available upon request. The SI includes supporting figures and table that provide additional data and discussion relevant to the main text, and is available at https://doi.org/10.1039/d5ra06956d.
