# Supplementary material for: Energy-efficient bifunctional CoBx/GDY catalyst for urea-assisted hydrogen production via electrochemical urea oxidation and hydrogen evolution
Source: RSC Adv. 2025 Oct 29;15(49):41253–9. doi: 10.1039/d5ra06956d (PMC12569999; doi:10.1039/d5ra06956d)
Supplement: RA-015-D5RA06956D-s001 [file RA-015-D5RA06956D-s001.pdf]

Supplementary Information (SI) for RSC Advances.
This journal is © The Royal Society of Chemistry 2025

Energy-efficient bifunctional CoB_x/GDY catalyst for urea-assisted hydrogen production via electrochemical urea oxidation and hydrogen evolution

Teng Liu,[a] Ting Wang,[a] Hao Niu,[a] Chunli Wang,[a] Zhenwei Wei,*[b] Jingjing Wang,[c] Xuepeng Yin,*[a] Shanmin Gao[a]

[a] T Liu, T. Wang, H. Niu, C. L. Wang, X. P. Yin, Prof. Dr. S. M. Gao

School of Chemistry & Chemical Engineering, Linyi University, Linyi 276000, PR China

[b] Z. W. Wei

School of Chemical Engineering and Technology, Tianjin University, Tianjin 300072, PR China

[c] J. J. Wang

College of Biological and Chemical Engineering, Qilu Institute of Technology, Jinan, 250200, PR China

*Corresponding author.

E-mail: yinxuepeng@lyu.edu.cn (X. P. Yin); weizw@tju.edu.cn (Z.W. Wei)

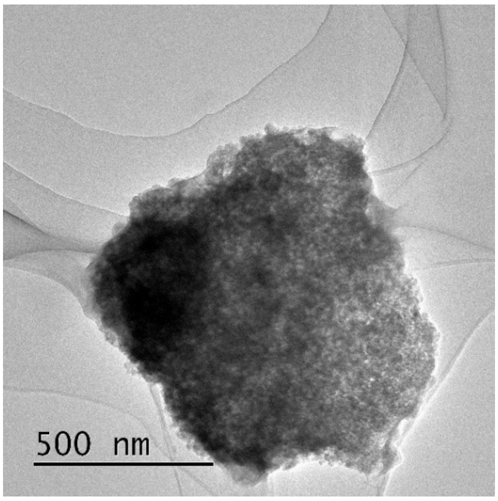

Figure S1. TEM image of CoB_x/GDY.

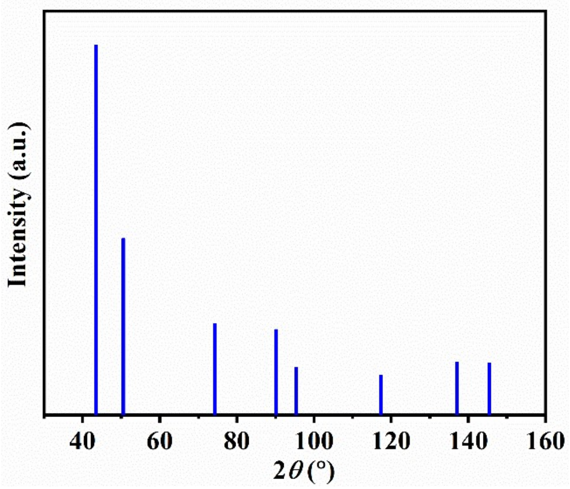

Figure S2. The standard diffraction pattern of metallic Cu (JCPDS card No. 65-9743).

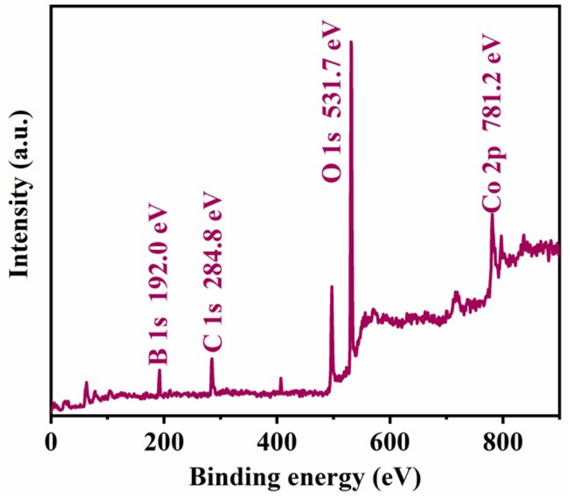

Figure S3. XPS wide survey spectrum for CoB_x/GDY.

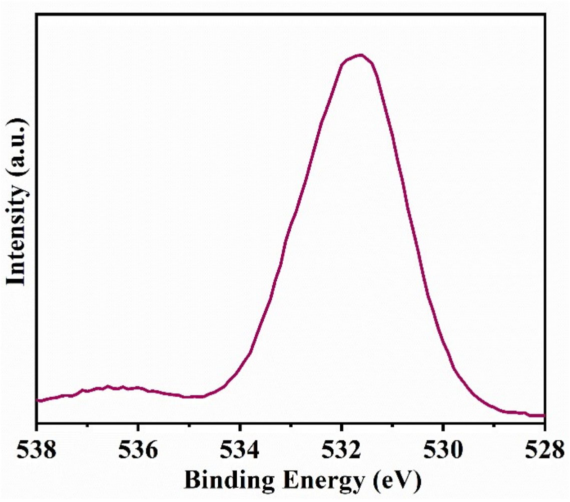

Figure S4. XPS spectrum of CoB_x/GDY in the O 1s region.

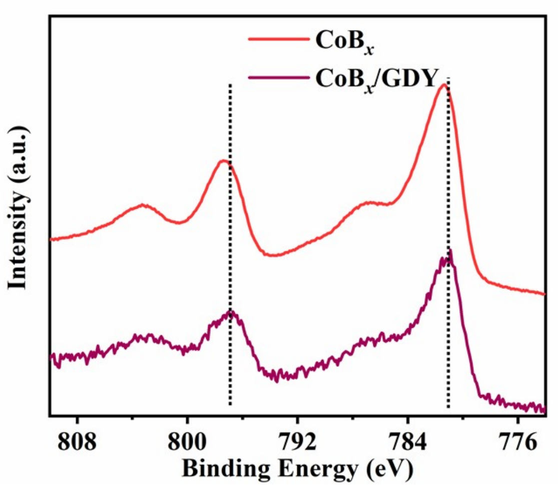

Figure S5. High-resolution Co 2p XPS spectra of CoB$_x$ and CoB$_x$/GDY.

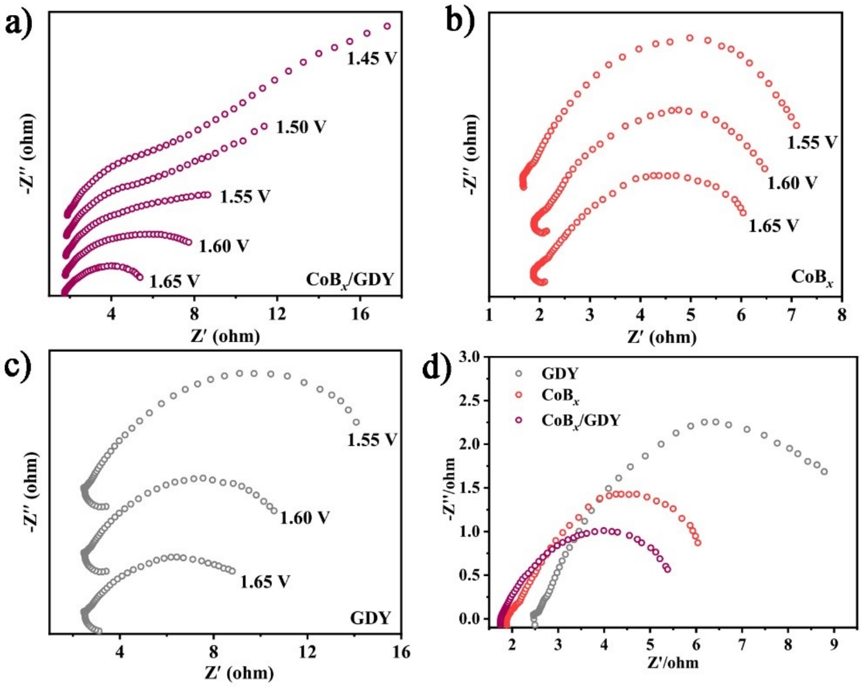

Figure S6. (a-c) Nyquist plots of CoB$_x$/GDY, CoB$_x$, and GDY at different operating potentials; (d) Nyquist plots of CoB$_x$/GDY, CoB$_x$, and GDY at 1.65 V.

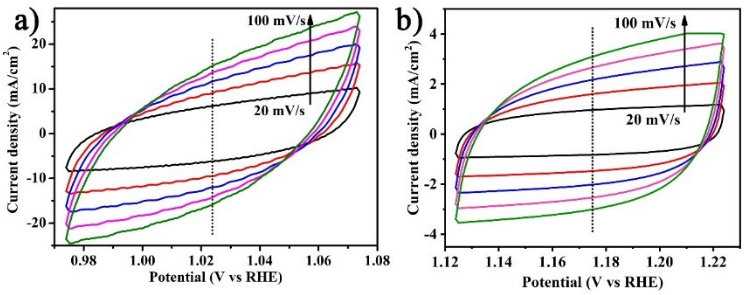

Figure S7. Typical CV curves for CoB$_x$/GDY (a) and CoB$_x$ (b).

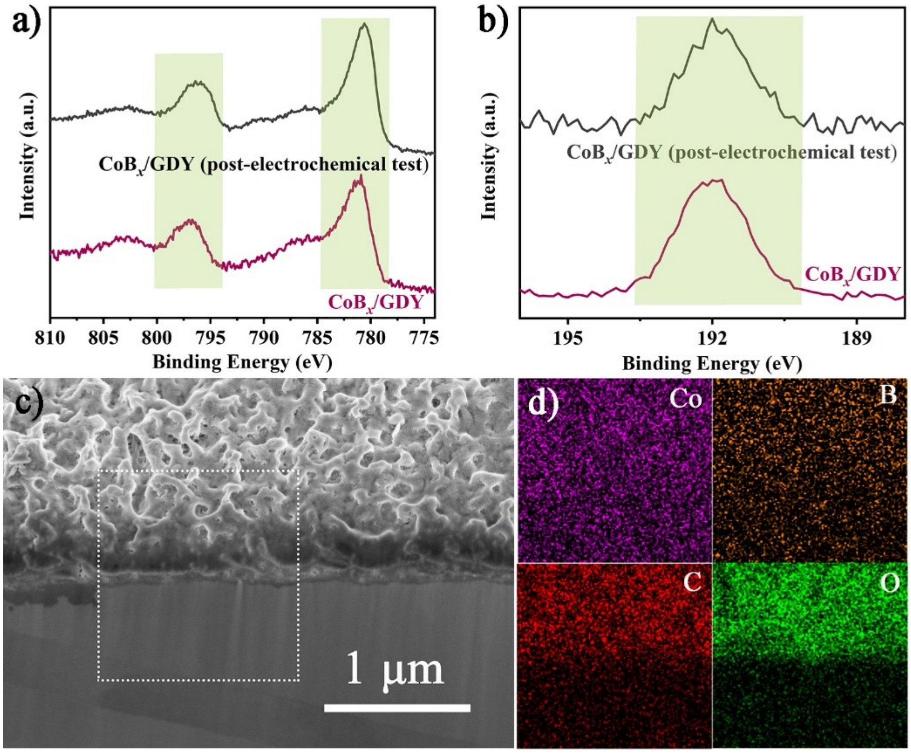

Figure S8. (a, b) Co 2p and B 1s XPS patterns of as-prepared CoB_x/GDY and the CoB_x/GDY after long-term electrolysis. (c, d) FIB-SEM image of CoB_x/GDY and the corresponding EDS elemental mapping (within the white-boxed region), showing the distribution of Co (purple), B (orange), O (green), and C (red).

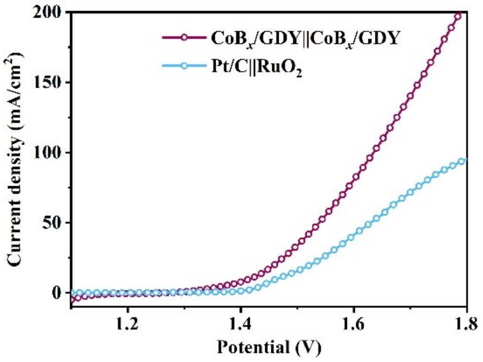

Figure S9. LSV curves of CoB_x/GDY||CoB_x/GDY and Pt/C||RuO_2 electrolyzers recorded in 1 M KOH containing 0.33 M urea.

Table S1. UOR performance of representative electrocatalysts (η_{10}, η_{50}, and η_{100}: potential at 10, 50, and 100 mA·cm^{-2}, respectively) and their corresponding Tafel slopes.

**Catalysts**	**Electrolyte**	**η (V)**	**Tafel Slope (mV·dec^{-1})**	**References**
CoB_x/GDY	1.0 M KOH + 0.33 M urea	η_{10}=1.26 V<br>η_{50}=1.41 V<br>η_{100}=1.49 V	58	This work
NiOOH/(LDH/α-FeOOH)	1.0 M KOH + 0.33 M urea	η_{10}=1.35 V<br>η_{50}=1.37 V<br>η_{100}=1.4 V	30.1	1
V2-$Ni(OH)_2$	1 M KOH+0.33 M urea	η_{100}=1.47 V	29.12	2
Ni-WO_x	1 M KOH + 0.33 M urea	η_{100}=1.40 V	39	3
Co–Ni–S@NF	1 M KOH + 0.33 M urea	η_{100}=1.35 V	29.6	4
Ru-Co DAS/NiO	1 M KOH + 0.33 M urea	η_{10}=1.29 V	23.3	5
CC/Ni-BDC@Co-PA	1 M KOH + 0.33 M urea	η_{100}=1.36 V	45	6
$Ni_{1.6}Co_{0.4}P$/C@HCNs	1 M KOH + 0.33 M urea	η_{10}=1.33 V	74.6	7
NF/P-$NiMoO_{4-x}$	1 M KOH + 0.33 M urea	η_{10}=1.36 V<br>η_{100}=1.38 V	19.3	8
O-NiMoP/NF	1 M KOH + 0.5 M urea	η_{100}=1.41 V	34	9
Co_xMo_yS-CC	1 M KOH + 0.5 M urea	η_{10}=1.31 V	—	10
Co(OH)F/NF	1 M KOH + 0.7 M urea	η_{10}=1.25 V	—	11
FeCo/$CoFe_2O_4$@NGC	1 M KOH + 0.33 M urea	η_{10}=1.32 V	42.31	12
Cu_3P-P/CF	1 M KOH + 0.33 M urea	η_{10}=1.43 V	33.1	13
HC-$Co(H_2PO_2)_2$	1 M KOH + 0.33 M urea	η_{10}=1.30 V<br>η_{10}=1.34 V	35	14
FeOOH@Co_3O_4	1 M KOH + 0.33 M urea	η_{10}=1.33 V	29	15
Fe_3O_4@FeOOH	1 M KOH + 0.33 M urea	η_{10}=1.38 V<br>η_{50}=1.42 V<br>η_{100}=1.46 V	28	16

$Co_2P/NiMoO_4/NF$	1 M KOH + 0.5 M urea	η_{10}=1.34 V	75	17
Ru/FeOOH@NFF	1 M KOH + 0.33 M urea	η_{10}=1.41 V	33	18
$Cu_3P@CuO_x$	1 M KOH + 0.33 M urea	η_{10}=1.37 V	29	19
$Ru_{0.4}CoP$-NC	1 M KOH + 0.33 M urea	η_{10}=1.30 V<br>η_{50}≈1.41 V<br>η_{100}≈1.47 V	97	20
$CoMoO_4/Co_9S_8$	1 M KOH + 0.5 M urea	η_{100}≈1.50 V	22	21

Reference：

1. M. Cai, Q. Zhu, X. Wang, Z. Shao, L. Yao, H. Zeng, X. Wu, J. Chen, K. Huang and S. Feng, *Advanced materials*, 2022, **35**.
2. H. Qin, Y. Ye, J. Li, W. Jia, S. Zheng, X. Cao, G. Lin and L. Jiao, *Advanced Functional Materials*, 2022, **33**.
3. L. Wang, Y. Zhu, Y. Wen, S. Li, C. Cui, F. Ni, Y. Liu, H. Lin, Y. Li, H. Peng and B. Zhang, *Angewandte Chemie International Edition*, 2021, **60**, 10577-10582.
4. Z. Xu, Q. Chen, Q. Chen, P. Wang, J. Wang, C. Guo, X. Qiu, X. Han and J. Hao, *Journal of Materials Chemistry A*, 2022, **10**, 24137-24146.
5. X. Zheng, J. Yang, P. Li, Z. Jiang, P. Zhu, Q. Wang, J. Wu, E. Zhang, W. Sun, S. Dou, D. Wang and Y. Li, *Angewandte Chemie International Edition*, 2023, **62**.
6. P. Li, W. Li, Y. Huang, Q. Huang, F. Li and S. Tian, *Small*, 2023, **19**.
7. S. Rezaee and S. Shahrokhian, *Nanoscale*, 2020, **12**, 16123-16135.
8. Y. Qiu, X. Dai, Y. Wang, X. Ji, Z. Ma and S. Liu, *Journal of colloid and interface science*, 2023, **629**, 297-309.
9. H. Jiang, M. Sun, S. Wu, B. Huang, C. S. Lee and W. Zhang, *Advanced Functional Materials*, 2021, **31**.
10. P. Li, Z. Zhuang, C. Du, D. Xiang, F. Zheng, Z. Zhang, Z. Fang, J. Guo, S. Zhu and W. Chen, *ACS applied materials & interfaces*, 2020, **12**, 40194-40203.
11. M. Song, Z. Zhang, Q. Li, W. Jin, Z. Wu, G. Fu and X. Liu, *Journal of Materials Chemistry A*, 2019, **7**, 3697-3703.
12. W. Chen, G. Qian, Q. Xu, M. Pan, L. Luo and S. Yin, *Electrochimica Acta*, 2021, **371**.
13. H. Shen, T. Wei, J. Ding and X. Liu, *Materials*, 2023, **16**.
14. K. Zhang, H. Yang, X. Han, S. Wang, X. Liu, T. Zou, J. Li, J. Zhang, H. Zhang and Y. Han, *Industrial & Engineering Chemistry Research*, 2024, **63**, 22403-22410.
15. Q. Zhang, M. Sun, M. Yao, J. Zhu, S. Yang, L. Chen, B. Sun, J. Zhang, W. Hu and P. Zhao, *Journal of colloid and interface science*, 2022, **623**, 617-626.
16. H. A. Bandal and H. Kim, *Journal of colloid and interface science*, 2022, **627**, 1030-1038.
17. M. You, S. Yi, G. Zhang, W. Long and D. Chen, *Journal of colloid and interface science*, 2023, **648**, 278-286.
18. P. Zhao, Q. Liu, X. Yang, S. Yang, L. Chen, J. Zhu and Q. Zhang, *Journal of colloid and interface science*, 2024, **673**, 49-59.
19. H. A. Bandal and H. Kim, *Applied Surface Science*, 2023, **622**.
20. L. Gu, Y. Li and A. D. Chowdhury, *Materials Today Chemistry*, 2023, **27**.
21. X. Du, C. Huang and X. Zhang, *International Journal of Hydrogen Energy*, 2019, **44**, 19595-19602.
